# Keyboard Data Protection Technique Using GAN in Password-Based User Authentication: Based on C/D Bit Vulnerability

**DOI:** 10.3390/s24041229

**Published:** 2024-02-15

**Authors:** Jaehyuk Lee, Wonbin Jeong, Kyungroul Lee

**Affiliations:** 1 Process Development Team, Fescaro, Suwon 16512, Republic of Korea; jaehyuk.lee@fescaro.com; 2 Department of Information Security Engineering, Mokpo National University, Muan 58554, Republic of Korea; goblebin@mokpo.ac.kr

**Keywords:** user authentication, keyboard data, password, machine learning

## Abstract

In computer systems, user authentication technology is required to identify users who use computers. In modern times, various user authentication technologies, including strong security features based on ownership, such as certificates and security cards, have been introduced. Nevertheless, password-based authentication technology is currently mainly used due to its convenience of use and ease of implementation. However, according to Verizon’s “2022 Data Breach Investigations Report”, among all security incidents, security incidents caused by password exposures accounted for 82%. Hence, the security of password authentication technology is important. Consequently, this article analyzes prior research on keyboard data attacks and defense techniques to draw the fundamental reasons for keyboard data attacks and derive countermeasures. The first prior research is about stealing keyboard data, an attack that uses machine learning to steal keyboard data to overcome the limitations of a C/D bit attack. The second prior research is an attack technique that steals keyboard data more efficiently by expanding the features of machine learning used in the first prior research. In this article, based on previous research findings, we proposed a keyboard data protection technique using GAN, a Generative Adversarial Network, and verified its feasibility. To summarize the results of performance evaluation with previous research, the machine learning-based keyboard data attack based on the prior research exhibited a 96.7% attack success rate, while the study’s proposed method significantly decreased the attack success rate by approximately 13%. Notably, in all experiments, the average decrease in the keyboard data classification performance ranged from a minimum of −29% to a maximum of 52%. When evaluating performance based on maximum performance, all performance indicators were found to decrease by more than 50%.

## 1. Introduction

As personal computers emerged and developed in the late twentieth century, the need to authenticate users accessing the system arose to protect information systems and personal information in computer systems [1]. To satisfy this need, user authentication technology emerged and continues to develop to this day [2]. Early user authentication technologies mainly used methods that were convenient to use and easy to implement, such as passwords that utilized information known to the user. Nevertheless, this password-based authentication technology can consist of short characters or numbers for the password. So, a security threat has arisen where the password is guessed or stolen when the user uses a password with low security or uses the same password in multiple services [3]. To solve these security threats, recently, in addition to knowledge-based authentication technology, ownership-based authentication technologies have been developed. These technologies include certificates and security cards that use information or devices owned by the user. So, device authentication technologies that authenticate by identifying information unique to the user device has also emerged [4].

The high proportion of security incidents caused by password exposures are caused by most services that provide user authentication adopting password-based authentication technology. Despite the emergence of various user authentication technologies, password-based authentication technology is much more efficient than the other authentication technologies in terms of difficulty in implementation and ease of use. Moreover, the biggest reason for using password-based authentication technology extensively is that applying or changing other authentication technologies may cause system compatibility problems. In addition, the adoption of additional authentication technologies incurs high costs [5]. The high costs are because many systems, including application, websites, and various services, have already adopted and use password-based authentication as their basic authentication technology.

As such, for various reasons, password-based authentication is still used as the main authentication technology in most systems. However, if the password is stolen by an attacker, serious damage such as personal information leakage, account hacking, and financial damage my occur. More seriously, secondary or tertiary damage may occur by attempted additional attacks due to leaked passwords. In order to prevent damage caused by password leakage as described above, security evaluation of password-based authentication technology is required. Furthermore, since passwords are inputted from the keyboard, research on technique to protect keyboard data is necessary. 

However, latest researches are actively conducted to efficiently steal keyboard data from an attacker’s perspective, rather than research from a defense perspective to protect keyboard data. Representative keyboard data attack techniques from an attacker’s perspective include WinProc substitution, message hooking, filter driver insertion, interrupt object substitution, IDT (Interrupt Descriptor Table) substitution, direct polling, exploitation of C/D bit vulnerability, and attacks utilizing the RESEND command [6,7,8,9]. Recently, to bypass the defense technique against attacks using C/D bit vulnerabilities [10], an attack technique that classifies random keyboard (hereinafter referred to as scan code) generated by the defender using a machine learning model has emerged. 

In this way, researches related to keyboard data are being conducted on new attack techniques from the attack side. In addition, researches to solve problems arising from the defense side and the development of defense techniques are essential to respond to continuously evolving attack techniques. This response helps to prevent damage caused by password attacks in advance. Therefore, in this article, to evaluate the security of password-based authentication technology and respond in line with evolving attack techniques, we propose a defense technique. In particular, this technique is against machine learning-based keyboard data classification attacks, the latest attack technique, using GAN [11]. The proposed technique using GAN is verified through experiments to effectively defend against attacks that steal keyboard data. Even if an attacker tries a keyboard data attack technique using data analysis by focusing on prior research on keyboard data attack techniques, the proposed defense technique works. 

The contributions of this study are as follows:

- This article points out the limitations of attack techniques based on C/D bit vulnerability. In addition, the article analyzes prior research on two keyboard data attack techniques using machine learning to overcome the above limitations. We also propose a GAN-based keyboard data protection technique using research experiments and datasets used in attack techniques. The proposed technique can protect the user’s authentication information more safely by decreasing the probability of keyboard data attack.
- We explore novelty by analyzing a keyboard data protection technique using CTGAN (Conditional Tabular GAN), which is used to generate two-dimensional data among generative adversarial networks called GAN.
- The maximum success rate of the machine learning-based keyboard data attack techniques in prior research was 96.7%, but as a result of applying the protection technique proposed in this article, the attack success rate was decreased by about 13%. Moreover, when in case of evaluating performance based on maximum performance, all performance indicators are superior, decreasing by more than 50%.

## 2. Prior Knowledge and Related Works

To successfully achieve the goal of this research, an understanding of the latest keyboard data attack and defense techniques is essential. Therefore, this section provides prior knowledge for understanding the keyboard data attack and defense techniques, and this prior knowledge includes the keyboard data transfer process, keyboard data attack and defense techniques. 

### 2.1. Keyboard Data Transfer Process

**Figure 1:**
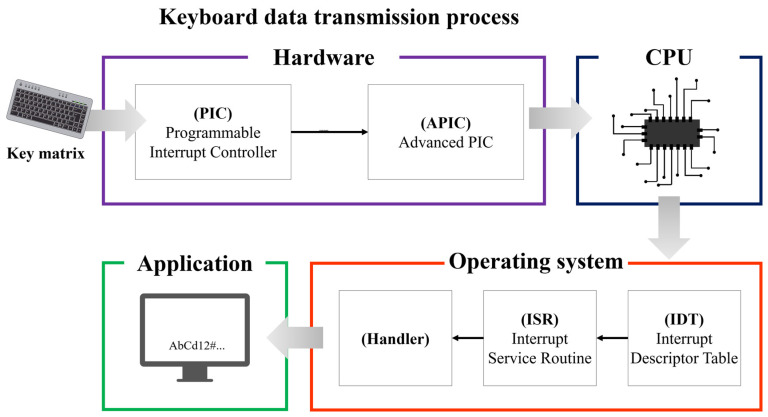
Keyboard data transfer process.

Keyboards are divided into PS/2 (Personal System/2) interface and USB (Universal Serial Bus) interface-based keyboards. In this article, a keyboard using the PS/2 interface was selected as the research object. The PS/2 interface is not used on desktops these days, but laptops connect the keyboard through the PS/2 interface. The keyboard data transfer process using the PS/2 interface is shown in Figure 1. 

The keyboard is an external input device that transfers the scan code corresponding to the key matrix inputted by the user to the CPU through the system bus, and the keyboard data is processed as an interrupt [12]. Therefore, the keyboard data inputted by the user is transferred to the interrupt controller to request interrupt processing. At this time, the destination controllers are the PIC (Programmable Interrupt Controller) and APIC (Advanced Programmable Interrupt Controller). When an interrupt request occurs, the PIC determines the source of the interrupt, performs a series of tasks to notify the operating system, and transmits the request to the APIC. The APIC generates an interrupt to the CPU based on the information received from the PIC. 

The CPU that receives the interrupt processes keyboard data through the interrupt table and handler. In the early days of computer development, the CPU performed all input and output processes on its own. However, for performance efficiency, the CPU was improved to prepare a separate interrupt table and handler and process it based on the prepared routine when an interrupt occurs [13]. Here, to efficiently handle interrupts, the tables and handlers prepared in advance were named interrupt table (IDT, Interrupt Descriptor Table), interrupt routine (ISR, Interrupt Service Routine), and interrupt handler, respectively. Afterwards, the result processed by the interrupt handler is passed to the application program, and the application program finally receives the keyboard data inputted from the keyboard device. Moreover, the operating system manages the transmission of keyboard data inputted by the user from the CPU to the application program, and at this time, the transmitted keyboard data is a scan code. In other words, the scan code is ultimately converted into a general character form within the operating system and transmitted to the application program, thereby displaying the keyboard data inputted by the user [14]. 

The PS/2 interface keyboard processes data inputted by the user through the transfer process described above. However, various vulnerabilities occur during this transfer process, and attack techniques using these vulnerabilities are mentioned earlier [6,7,8,9]. 

### 2.2. Related Works

In this subsection, we describe researches on keyboard data attack and defense techniques that are related to the present research. The ultimate goal is to select keyboard data attacks caused by C/D bit vulnerability as a core technique and propose defense technique to counter this attack technique. Accordingly, we focus on attacks and defenses using C/D bit vulnerability. To understand this attack technique, direct polling-based attack technique, random scan code generation-based defense technique, and C/D bit vulnerability-based attack technique are described. 

#### 2.2.1. Direct Polling-Based Attack Technique 

Direct polling-based attack technique is an attack technique at the hardware level that is attempted at a layer lower than the operating system. Polling refers to the operation of the operating system in a computer system to periodically read the status register of an input and output device [15]. Namely, direct polling is used to steal keyboard data as soon as the user inputs a key from the keyboard device. This stealing is achieved by preempting and checking the state in which keyboard data has been inputted by periodically reading keyboard status information. The reason why such an attack is possible is found to be that the attacker takes precedence over the operating system in obtaining status information. Notably, a separate atomic operation is prepared at the operating system level to prevent loss of keyboard data. 

Attackers attempt direct polling-based attacks by periodically reading the keyboard status using the atomic operation of the operating system. Additionally, direct polling-based attack technique is not only used by attackers, but the operating system also processes keyboard data in the same way. This causes a limitation that makes it difficult to determine whether an access is from the operating system or an attacker. For this reason, this attack results in serious damage and the system does not prevent attacks that steal keyboard data. In addition, there is no clear defense technique against direct polling attacks, and even if an attack occurs, it is impossible to detect whether the attack occurred. Table 1 shows the status register configuration of the keyboard controller used in direct polling-based attack technique [16,17]. 

In general, keyboard data communication is accomplished through the keyboard controller (8259A) inside the host, and data is transmitted and received through separately provided ports. Here, the separately provided ports are the control and data ports of the fourth bit, and the control port is composed of 0x64 and the data port is composed of 0x60. Moreover, each port has separate input and output buffers, and the keyboard and host communicate with each other through these buffers. So, reading the control port and data port means obtaining transmitted keyboard status information and keyboard data, respectively. Conversely, when a command code is passed to the control and data ports, the command corresponding to the code is sent to the keyboard and controller and the command received from the keyboard and controller is executed. 

**Figure 2:**
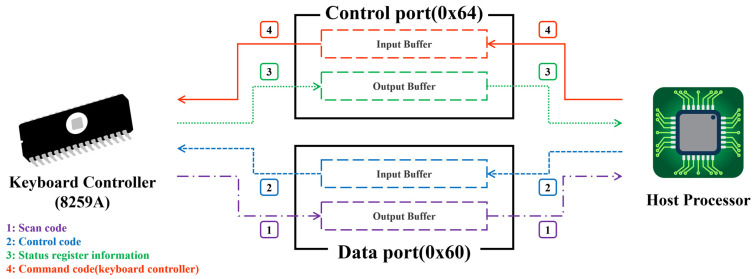
Example of communication flow between the keyboard controller and host.

Meanwhile, OBF means the output buffer is full. Likewise, IBF means the input buffer is full. Namely, by reading the OBF of the control port, the attacker confirms that data has been transmitted from the keyboard. This status means that the user inputted a key on the keyboard device, and it was transmitted to the controller. In the end, if OBF is set, data is transmitted from the keyboard, so reading the data port means stealing keyboard data. As described above, data is communicated between the keyboard and the host, and this is diagrammatically shown in Figure 2.

#### 2.2.2. Defense Technique Based on Random Scan Code Generation

As mentioned above, direct polling-based attack technique is an attack that steals data by checking the keyboard input status while the user inputs keys through the keyboard [9]. To respond to this direct polling attack, various novel defense techniques have been researched. Among them, the representative defense technique is a defense technique based on random scan code generation [10]. This technique is an attack that causes confusion to the attacker by having the defender generate a random scan code. This random scan code makes it difficult for the attacker to distinguish whether the stolen scan code is the actual scan code inputted by the user. This technique uses specific commands among the control commands of the keyboard controller. The main commands of the keyboard controller are shown in Table 2.

Analyzing the main control commands of the keyboard controller, the 0xD2 control command is a command that writes a value to the keyboard output buffer. This control command transmits the scan code you want to generate to the output buffer, generates the scan code received from the controller, and causes an interrupt to the CPU. Accordingly, it is possible to generate a random scan code by utilizing this command. Therefore, the keyboard data defense technique based on random scan code generation prevents the attacker’s scan code stealing attack by having the defender randomly generate a scan code using the 0xD2 command. This defense technique is assumed to operate in separate security software implemented by the defender, and the detailed operation process is shown below. 

Step 1. The security software generates a random scan code and then sends the 0xD2 command to the control port to notify the keyboard controller to generate a random scan code. 

Step 2. After transmitting the 0xD2 command, when the generated scan code is transmitted to the data port, the keyboard controller generates a keyboard interrupt according to a series of prepared procedures. 

Step 3. The generated interrupt calls the handler according to the interrupt routine defined in the operating system and transmits the scan code to the security software. 

Step 4. The security software compares the received scan code with the generated scan code. If the scan code is the scan code generated by the security software, it is not a scan code inputted by the user, so it returns to Step 1 and repeats the previous process. If the scan code is not generated by the security software, the scan code is processed based on the scan code inputted by the user. 

**Figure 3:**
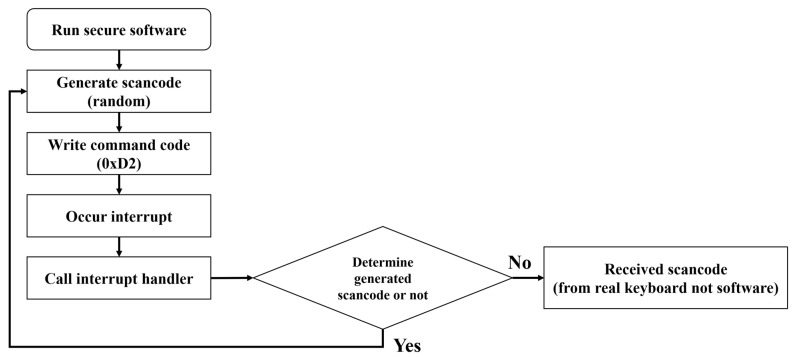
Defense technique operation process based on random scan code generation.

In the keyboard data defense technique based on random scan code generation, the defender is able to distinguish scan codes inputted by the user even if random scan codes are continuously generated. On the contrary, an attacker collects both the random scan code generated by the security software and the scan code inputted by the user. However, the attacker cannot steal the scan code inputted by the user, because among the two scan codes, there is no information about the random scan code generated by the security software. Following this process, this defense technique emerged as an efficient way to respond to direct polling-based attack technique. The operation process of the random scan code generation-based defense technique is shown in Figure 3.

#### 2.2.3. C/D Bit Vulnerability-Based Attack Technique

The random scan code generation-based defense technique is a defense technique that safely protects keyboard data by randomly generating scan codes in security software. This technique works even if an attacker carries out a keyboard data attack such as the one using the direct polling-based attack technique. Although this defense technique effectively responds to direct polling-based attack technique, it does not guarantee security due to the following problems [18,19]. The security vulnerability that arises from random scan code generation-based defense technique is a vulnerability that arises from the C/D bit. So, a novel keyboard data attack technique that exploits this vulnerability has been researched. 

The C/D bit indicates the control port or data port for the purpose of checking from which port the received command was transmitted. For example, if data was received through the control port, C/D is set to 1 (TRUE), and if data was received through the data port, C/D is set to 0 (FALSE). Therefore, from the attacker’s perspective, the C/D bit is periodically observed. Notably, if OBF occurs after the C/D bit is set to 0, this serves as the basis for determining that the security software has randomly generated a scan code to neutralize the keyboard data attack. This means that for the security software to generate scan code, it must transmit the 0xD2 command to the control port and transmit a random scan code to the data port, thereby the corresponding scan code is generated. Therefore, in this process, since data is written to the data port at the end, the C/D bit must be set to 0.

On the other hand, if OBF occurs after the C/D bit is set to 1, the basis for determining that the data is actual keyboard data inputted from the user is revealed. To distinguish these random and actual scan codes, the attacker sends a command to set the C/D bit to 1 to the control port, thereby always fixing the C/D bit to 1. Afterwards, if OBF occurs after the C/D bit is set to 1, the security software has not generated a random scan code, so it can be determined as actual keyboard data input by the user. Based on these results, the attacker periodically monitors the C/D bit to classify the random scan code generated by the defense technique and the actual scan code. This is a keyboard data attack technique utilizing the C/D bit vulnerability.

### 2.3. Prior Researches

Nevertheless, there were limitations to the C/D bit vulnerability-based attack technique, and by explaining two prior researches to overcome these limitations, we help understand the proposed keyboard data defense technique using GAN.

#### 2.3.1. Research on Keyboard Data Attack Using Machine Learning

This prior research introduced an attack technique to steal keyboard data using machine learning from the attacker’s perspective [18]. First, the C/D bit vulnerability-based attack technique is a technique that exploits the vulnerability of classifying random keyboard data generated by security software and actual keyboard data inputted from the keyboard by the C/D bit value. For this purpose, the C/D bit and OBG bit must be monitored periodically, and all inputted scan codes must be preempted. To meet these demands, system overload occurs due to monitoring and preemption, and this overload appears to defenders as a clue to detect the keyboard data attack. Therefore, to overcome these problems with the attack, this prior research proposed a novel keyboard data attack technique that does not cause abnormal behavior such as system overload by using machine learning models. 

Moreover, to improve attack performance more efficiently, three datasets were constructed to select machine learning models according to the situation. Each dataset consisted of a different number of randomly generated random scan codes and actual scan codes inputted from a real-world keyboard. Moreover, by defining various characteristics for learning, the dataset with the best performance was derived through experiments. Each constructed dataset is shown in Table 3, and rather than using a single machine learning model, the model with the best performance was verified through experiments. These experiments were performed by learning using KNN (K-Nearest Neighbors) [17], Logistic Regression [20], Decision Tree [21], Random Forest [22], GBRT (Gradient Boosting Regression Tree) [23], SVM (Support Vector Machine) [24], and MLP (Multi-layer Perceptrons) [22], which are various machine learning models. 

Among the three datasets in Table 3, the experiment using the second dataset (Exp. 2.) showed the highest performance. To summarize the overall experimental results, the attack success rate for keyboard data classification was found to be as low as 90.4% and as high as 96.2%. As a result, it was verified that using the machine learning-based keyboard data attack technique proposed in prior research solves the problems arising from the C/D bit vulnerability-based attack technique. The machine learning-based keyboard data attack technique also steals actual keyboard data with excellent performance. 

#### 2.3.2. Research on Machine Learning-Based Keyboard Data Attack through Feature Expansion

In this subsection, we describe the second prior research, research on improving keyboard data attack performance through feature expansion [19]. The goal of this research is to further improve the keyboard data attack performance of the first prior research by expanding the features. Specifically, to analyze the security threats of IoT (Internet of Things) devices that are increasing exponentially according to the fourth industrial revolution, attack scenarios that can occur in IoT services are derived. From a technical perspective, this prior research expanded the keyboard data elapsed time and scan code, which are the characteristics defined in the first prior research. So, the difference in keyboard data elapsed time and the distance to the scan code are defined as new features to improve keyboard data attack performance. Here, the keyboard data elapsed time refers to the time when random keyboard data generated by security software or actual keyboard data delivered by the actual user is inputted. Further, the scan code refers to keyboard data. 

Based on the above information, we assumed that random keyboard data and actual keyboard data will be more effectively classified by applying Manhattan distance and Euclidean distance to the keyboard data elapsed time difference and scan code. The Manhattan distance is a measurement of the distance between two coordinates by converting the elapsed time and scan code into coordinates for measuring the distance between two coordinates which are the previous and current coordinates. The Euclidean distance measured the distance between two coordinates based on the initial coordinates (0, 0) and the previous and current coordinates. The features expanded from the first prior research are shown in Table 4.

Analyzing the dataset used in the second prior research, we find that the dataset of Exp. 1. is not actually used for keyboard data classification. However, since the feature that showed the highest performance is in the first dataset, we constructed this dataset for the purpose of comparing performance with the proposed technique. So, the actual datasets consist of six datasets from Exp. 2. to Exp. 7. The performance evaluation results according to the six constructed datasets are shown in Table 5, Table 6, and Table 7, and only the highest performance is shown to summarize.

We analyze the attack success rate for improved keyboard data classification achieved by defining expanded features. Compared to first prior research, the performance of the second prior research with expanded features was improved by a minimum of 10.6% and a maximum of 16.1% based on the average of overall performance. In addition, a high classification performance of approximately 96.7% based on classification accuracy was shown with the expanded features. As a result, this second prior research that expanded the features verified that it is possible to steal actual keyboard data with even more improved performance than the first prior research. These results will be used as an indicator to compare and analyze the performance of the defense technique proposed in this article.

## 3. Proposal of A Keyboard Data Defense Technique Using GAN

In this section, based on the analysis results of prior researches, we propose a defense technique to counter a keyboard data attack technique. The proposed technique is from a defense research to safely protect keyboard data from the machine learning-based keyboard data attack technique. This research uses a GAN artificial intelligence model to confuse attackers in keyboard data classification attacks. To propose a keyboard data defense technique using GAN, we describe the basic idea proposed and the result related to the verification of the possibility of its defense against the attack technique. This verification is given in the “Proposed defense technique methodology” section focused on the methodology for the proposed defense technique. The “Keyboard data defense system configuration” section describes in detail the configuration of an actual defense system using the proposed methodology. Based on this configuration, the optimal keyboard data defense technique is ultimately derived.

### 3.1. Proposed Defense Technique Methodology

This subsection explains the basic idea proposing the use of GAN in keyboard data attack defense technique by explaining the perspectives of attackers and defenders. We also verify the feasibility of keyboard data defense based on GAN by comparing the distribution of random keyboard data and actual keyboard data in the prior research datasets and the datasets of the proposed technique.

#### 3.1.1. Basic Idea

To understand the basic idea of the proposed defense technique methodology, it is first necessary to understand it from the perspectives of both the attacker and the defender. The ultimate goal of the attacker is to steal the actual keyboard data inputted by the user to neutralize password-based user authentication. Likewise, the final goal of the defender is to protect the actual keyboard data from being stolen by the attacker. So, a conventional defense technique against the attack is for defenders to interfere with keyboard data classification by attackers by generating random keyboard data in a security software. Despite these obstacles, attackers use machine learning to classify actual keyboard data with a very high success rate. This classification is based on the characteristics that appear in keyboard data actually inputted by the user and artificially generated random keyboard data, which is the latest attack technique against prior defense techniques. This is the perspective of the attacker and defender in this article. 

Since attackers use machine learning to steal actual keyboard data, defenders need novel defense technique to prevent actual keyboard data from being stolen by classifying random and actual keyboard data. The idea of a defense technique that satisfies these requirements is GAN, and in this article, we propose a methodology to neutralize machine learning-based keyboard data attack technique using GAN. To understand the proposed methodology, an understanding of GAN is necessary. 

GAN is one of the machine learning models first introduced in 2014 [25]. In this model, two different systems, consisting of a generator and a discriminator, learn while competing with each other. GAN is mainly used in the field of image restoration and generation. For example, assuming that there is some image data, among the two systems, the generator is responsible for generating fake image data similar to real data. On the contrary, the discriminator serves to distinguish between fake image data created by the generator and existing real image data. When this process is repeated, the generator and discriminator compete with each other, resulting in improved performance. Ultimately, the generator’s ability to create fake images improved to the point where the discriminator cannot distinguish between real images and the fake images created by the generator. Finally, learning ends when the discriminator’s discrimination accuracy reaches 50%. 

In summary, GAN is a machine learning model that generate fake behavior with characteristics similar to the real thing or generates fake data that is likely to exist in reality. Therefore, if random keyboard data similar to the actual one is generated through GAN, it is expected to decrease the success rate of attacks that steal actual keyboard data. This decrease is because even if keyboard data attack technique uses machine learning, the features defined to classify actual keyboard data and random keyboard data become ambiguous. This is why this article applied GAN to propose a defense technique to protect keyboard data and proposed a novel methodology to protect keyboard data.

#### 3.1.2. Verification of Keyboard Data Defense Feasibility Based on GAN

In this subsection, the possibility of keyboard data defense is verified based on the methodology of the keyboard data defense technique described in “Section 3.1.1. Basic idea”. The proposed defense technique uses GAN to generate random keyboard data with characteristics similar to the real thing to cause confusion during the attack. Therefore, to verify the feasibility of defense using GAN, it is necessary to generate random keyboard data using GAN. 

Typically, GAN models are used for image generation and restoration. However, since the defense technique proposed in this article generates random keyboard data rather than image data, it is not appropriate to use GAN model. For keyboard data defense technique, the data generated by the GAN model are elapsed time and scan code. Ultimately, GAN model that generates two-dimensional data must be selected, because the generated data consists of two-dimensional column data, such as elapsed time and scan code. 

**Figure 4:**
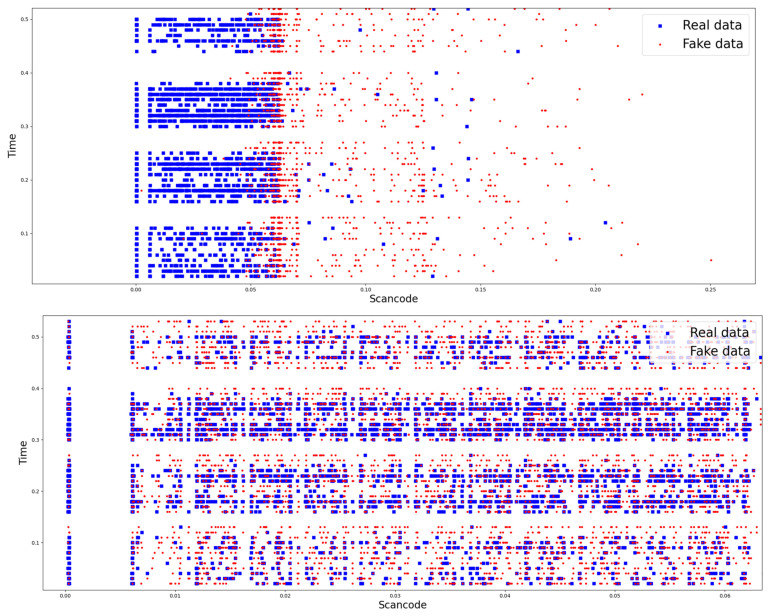
Results of comparing the distribution of random keyboard data and actual keyboard data in the prior research dataset and the dataset of the proposed technique.

So, this article selected CTGAN, which is widely used to generate two-dimensional data in the fields of data analysis and machine learning [26]. Based on the selected model, the similarity between random keyboard data generated from GAN model and actual keyboard data was found to imply the feasibility of defense. To compare similarity, the distribution of random and actual keyboard data for the dataset used in the keyboard data attack technique using machine learning, which is a prior research, is shown in Figure 4. The figure also shows the distribution of random and actual keyboard data generated from the GAN model.

As shown in the figure, the distribution map shown in the figure above is the distribution map of random and actual keyboard data for the dataset used in prior research. That is, the dataset used in the keyboard data attack technique using machine learning before applying the GAN model. The distribution map below shows the distribution of random and actual keyboard data after applying the GAN model. To explain the distribution map in detail, the distribution map before applying GAN models shows a clear difference in the distribution of data inputted from the actual keyboard (blue dots) and data generated from security software (red dots). On the other hand, the distribution of map after applying GAN model shows that the distributions of blue and red dots are mixed together to the extent that it is difficult to distinguish between the two data. 

If the distribution result is interpreted from a machine learning perspective, the distribution map before applying the GAN model can distinguish between random keyboard data and actual keyboard data. This means that the two data can be easily classified through machine learning. In fact, the results showed that actual keyboard data was classified with 96.7% accuracy before applying GAN. This result is believed to be due to the fact that random keyboard data generated by security software is generated every 50 ms and appears to have significantly different characteristics from actual keyboard data directly inputted by a human. However, since the distribution map after applying GAN model appears to be at a level where it is difficult to distinguish between random keyboard data and actual keyboard data. So, it is expected that keyboard data classification through machine learning will be difficult in this situation. Consequently, we believe that the keyboard data defense technique using GAN proposed in this article will effectively neutralize the keyboard data attack technique using machine learning.

### 3.2. Keyboard Data Defense System Configuration

In “Section 3.1.1. Basic idea” and “Section 3.1.2. Verification of keyboard data defense feasibility based on GAN”, we established a methodology using GAN, which is the basic idea of this article. Also, the feasibility of keyboard data defense was verified based on the established methodology. Analyzing the verification results, it was found that the random keyboard data generated using GAN was very similar to actual keyboard data to compare with the dataset used in prior keyboard data attack technique. 

**Figure 5:**
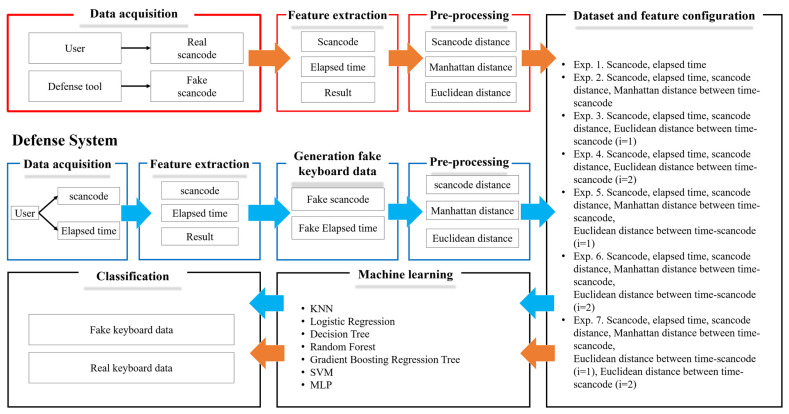
Prior attack system and defense system configuration diagrams.

Based on these verification results, this section describes the configuration of a defense system for applying keyboard data defense technique using GAN in password-based authentication. The defense system to protect keyboard data mainly consists of seven steps, in order: “1. Data collection step, 2. Feature extraction step, 3. Random keyboard data generation step, 4. Data preprocessing step, 5. Experiment and dataset configuration step, 6. Machine learning step, and 7. Classification step”. To more intuitively show and compare the configuration of the prior attack system and experiment configuration of the defense system, the overall configuration diagram of the prior attack system and the defense system are shown in Figure 5.

#### 3.2.1. Data Collection Step

In the prior attack and defense system configuration diagrams shown in Figure 5, blue arrow represents the configuration of the defense system, and amber arrow represents the prior attack system. The attack system collects both the scan codes, which are the actual keyboard data inputted by the user and the random scan code generated by the security software. After configuring all the collected random and actual keyboard data into a dataset, the two data are classified using machine learning models to steal the actual keyboard data. To respond to these attacks, the defense system, like the attack system, requires a data collection step to utilize a machine learning model. This step is to collect data corresponding to the actual scan code and elapsed time, which is the keyboard data inputted by the user. Here, the purpose of collecting only data on scan code and elapsed time is to collect the same information to compare performance based on the same environment and data. Also, notably, the key features used in prior attack system are scan code and elapsed time.

#### 3.2.2. Feature Extraction Step

In the feature extraction step, features are extracted from keyboard data to analyze data to protect keyboard data based on the data collected in the previous data collection step. Data that can be defined as features from the keyboard data collected in the previous step are scan code and elapsed time, which are the same as the data collected in the “Section 3.2.1. Data collection step”. However, to use data on scan code and elapsed time for the purpose of data analysis, a separate step of extracting features from the collected data is necessary. 

The scan code refers to the actual keyboard data generated when the user input through the keyboard. The scan code data transmitted in this process consists of one hexadecimal byte rather than letters or numbers like in the keyboard. Also, the range of the scan code is expressed from 0 to 255 in decimal, so to learn more easily with machine learning, the scan code is normalized to have a range of 0 to 1 and defined as a feature. Elapsed time refers to the time for which the current keyboard data was collected, which is the difference between the time when the previous keyboard data was collected and the time the current keyboard data was collected. This elapsed time is defined as a feature for machine learning classification. Finally, using the two defined features, scan code and elapsed time, define a binary label (0, 1) consisting of true or false indicating actual keyboard data and random keyboard data to be used for data classification in the machine learning model. In the labels defined in this article, 0 means random keyboard data, and 1 means actual keyboard data. 

#### 3.2.3. Random Keyboard Data Generation Step

The random keyboard data generation step is to generate realistic random keyboard data using GAN based on data on the actual scan code and elapsed time collected in the previous step. The methodology for the defense technique proposed in this article is intended to counter prior machine learning-based keyboard data classification attacks. So, the ultimate goal of this methodology is to prevent random keyboard data and actual keyboard data generated by security software from being classified through machine learning. To do this, it is required to generate random keyboard data with similar characteristics to actual keyboard data. This similarity is to cause confusion so that the attacker does not classify random keyboard data and actual keyboard data from the attacker’s perspective. According to these requirements, this step is the most essential function of the defense technique proposed in this article. 

To generate random keyboard data similar to actual keyboard data, it is necessary to select GAN model. In this article, as verified in “Section 3.1.2. Verification of keyboard data defense feasibility based on GAN”, a CTGAN model suitable for two-dimensional data generation in the field of data analysis and machine learning was selected. Based on the selected model, data collected through the “Section 3.2.1. Data collection step”, and “Section 3.2.2. Feature extraction step” are used to generate random keyboard data similar to the real thing. Specifically, since the goal is to generate random keyboard data similar to the real one, random keyboard data is generated based on the actual keyboard data inputted by the user. An example of the actual keyboard data used as input to the CTGAN model and the generated random keyboard data is shown in Table 8.

In the table, the left column is the actual keyboard data used as input to generate the random keyboard data, and the right column is the random keyboard data generated by the GAN model. Analyzing the generated data, it was found to be so similar to the actual keyboard data that it was difficult to classify which data is actual keyboard data and which data is random keyboard data generated from GAN model. Nevertheless, for more accurate classification, performance evaluation of the generated random keyboard data is necessary. 

In general, performance evaluation of GAN models includes the IS (Inception Score) method, which measures the classification performance of the image generation model. Also, the FID (Frechet Inception Distance) method [27], which measures the difference between the image generated by the model and the actual image, is used for performance evaluation. Finally, the PPL (Perceptual Path Length) method [28], which measures image consistency, is also used in performance evaluation. However, a performance evaluation method other than the existing performance evaluation methods is required, because the data used in this article is two-dimensional data consisting of numbers rather than images. 

The performance evaluation method for the two-dimensional data generation model is the KS (Kolmogorov-Smirnov Test) method [29], which verifies the difference between the data distribution generated by the model and the actual data distribution. Likewise, the KL-divergence (Kullback-Leibler divergence) method [30], which evaluates quantitative performance by numerically implementing the KS-Test is also used. Finally, a model testing method based on generated data that measures the results by training another machine learning model using the data generated by the trained model is also used for the performance evaluation. 

In this article, among these various performance evaluation methods, a generative data-based model testing method was selected to evaluate the performance of GAN model. Notably, two elements were added to propose a more efficient keyboard data defense technique. The first element is the choice of whether to add random keyboard data generated from GAN model to the dataset used in the existing attack, or to delete and replace the existing random keyboard data. The second element is to derive the optimal number of learning iterations (epoch) to derive the performance difference according to the hyper parameters of the CTGAN model. Including these two elements, the performance of the CTGAN model was evaluated. 

**Figure 6:**
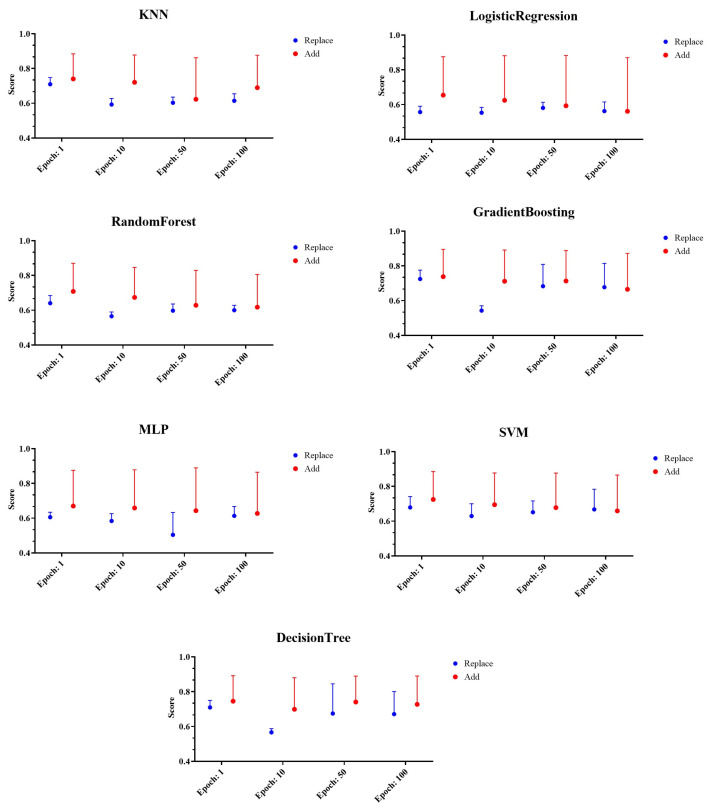
Example of performance evaluation results of random keyboard data generated from CTGAN model.

For performance evaluation, the performance of machine learning models such as KNN, logistic regression, decision tree, random forest, GBRT, SVM, and MLP was evaluated based on the selected generated data-based model testing method. The performance evaluation results for random keyboard data generated from CTGAN model are shown in Figure 6.

The figure shows the performance evaluation results according to the deletion and replacement of the existing dataset and the performance results according to the number of iterations of GAN model. Meanwhile, the scores on the Y axis are accuracy, precision, recall, F1-score, and AUC performance indicators shown on average, and the error rate was expressed graphically. Analyzing the results, we found evidence that all machine learning models except MLP showed the lowest performance at the number of iterations of 10. In addition, the method of replacing existing random keyboard data with random keyboard data generated by GAN model was showed to have the lowest performance. Since the proposed technique aims to decrease the success rate of prior keyboard data classification attacks, the existing data replacement method with the lowest performance, was found to be the most suitable.

#### 3.2.4. Data Preprocessing Step

The data preprocessing step is to expand the features of the random keyboard data, which is the random scan code and random elapsed time. This step is also to preprocess the keyboard data generated in the “Section 3.2.3. Random keyboard data generation step” to make it suitable for machine learning classification. The main purpose of the technique proposed in this article is not to improve the performance from the perspective of attacks that classify keyboard data using machine learning. Instead, data is preprocessed in the same way as prior researches to compare and evaluate the performance of the proposed defense technique with prior researches corresponding to prior attack techniques. 

In prior researches, to improve the performance of machine learning models, the distance between the previous scan code and the current scan code and the distance between time and scan code were measured. A total of three methods were used for this purpose. These methods find the distance between scan codes, distance measured through time-scan code Manhattan distance, and distance measured through time-scan code Euclidean distance and define them as features. First, the first method, the distance measurement method between scan codes, is a method of calculating the difference in distance between the previously collected scan code and the currently collected scan code. At this time, the scan code has values between 0 and 1, as normalized in the “Section 3.2.2. Feature extraction step”. To improve the learning efficiency of the machine learning model, the absolute value was applied to have only positive values above 0. The distance measurement between scan codes is shown in Equation (1), where SC is the current scan code and SP is the previous scan code.

(1) scan code distance=|SC−SP|                           

The time-scan code Manhattan distance measurement method measures the characteristics of both elapsed time and scan code to improve classification performance for keyboard data. To explain in detail, the scan code has the characteristic of having sequential values according to the arrangement of the keyboard keys. In addition, the elapsed time was set to a 50 ms period for random keyboard data generated by the security software. The distance is measured to reflect these characteristics, because actual data inputted from a physical keyboard has aperiodic characteristic. Namely, the time-scan code Manhattan distance measurement method converts the elapsed time and scan code into coordinates, and measures the distance between the previous coordinates and the current coordinates using the Manhattan distance. The time-scan code Manhattan distance is shown in Equation (2), where T is the elapsed time and S is the scan code.

(2) $$Manhattan\;distance\;\left(T,\;S\right)=\overset{n}{\underset{i=1}{\sum }}\left|Ti-Si\right|\;\;\;\;\;\;\;\;\;\;\;\;\;\;\;\;\;\;\;\;\;\;\;\;$$

The time-scan code Euclidean distance measurement method is similar to the Manhattan distance measurement method. However, the Euclidean distance measurement method uses the Euclidean distance rather than the Manhattan distance, and measures the distance in two ways. The first method is to convert the elapsed time and scan code into each X and Y coordinates and measure the distance from the initial coordinates (0, 0) to the current coordinate. The i value in this case is 1. The second method is the same as converting the elapsed time and scan code to each X and Y coordinates, but measures Euclidean distance from the previous coordinates to the current coordinates. The i value in this case is 2. The time-scan code Euclidean distance is shown in Equation (3), where T is the elapsed time and S is the scan code.

(3) $$Euclidean\;distance\;\left(T,\;S\right)=\sqrt{\overset{n}{\underset{i=1}{\sum }}{\left|Ti-Si\right|}^{2}}\;\;\;\;\;\;\;\;\;\;\;\;\;\;\;\;\;\;\;\;\;\;\;\;\;\;\;\;\;\;$$

To summarize the distance measurement methods, an attack system in prior researches was defined as a feature by measuring elapsed time and scan code distance using the three preprocessing methods described above. Accordingly, in this article, to compare and evaluate the performance with prior researches in the same environment and dataset, data is preprocessed targeting random scan codes and elapsed time data generated in the “Section 3.2.3. Random keyboard data generation step”. Based on the data generated in the third step, the random keyboard data generation step, the results of data preprocessing are shown in Table 9.

The preprocessed data, including the distance between scan codes, time-scan code Manhattan distance, time-scan code Euclidean distance (i = 1), and time-scan code Euclidean distance (i = 2), are shown in the table. These data preprocessed in this step are used as features in the subsequent machine learning step.

#### 3.2.5. Experiment and Dataset Configuration Step

The experiment and dataset configuration step is the step of configuring datasets for machine learning based on the features expanded in the previous “Section 3.2.2. Feature extraction step”, and “Section 3.2.4. Data preprocessing step”. Moreover, the dataset configured at this step is used in experiments to evaluate performance. To compare and evaluate the performance of two prior researches and the defense technique proposed in this article, the same dataset is used. 

Specifically, based on the basic features of scan code and elapsed time, distance between scan codes, time-scan code Manhattan distance, time-scan code Euclidean distance (i = 1), and time-scan code Euclidean distance (i = 2) were expanded as features. So, six features were used to configure datasets for the experiment. Moreover, the configured datasets are prevented from having an inappropriate impact on machine learning performance. For this prevention, each dataset was configured to have a different ratio of actual keyboard data and random keyboard data generated from GAN model. The configuration of the dataset for machine learning is shown in Table 10.

#### 3.2.6. Machine Learning Step

The machine learning step is the step of learning a model that classifies actual keyboard data and random keyboard data using a machine learning model based on the dataset configured in the “Section 3.2.5. Experiment and dataset configuration step”. In general, machine learning models are very diverse, and depending on the characteristics of the data, the machine learning model suitable for classification may be different. So, to select a machine learning model with the best performance in terms of keyboard data classification, evaluation of the performance derived from various models is required. To meet these requirements, prior researches used various machine learning models such as KNN, logistic regression, decision tree, random forest, GBRT, SVM, and MLP. For consistent comparison with prior researches, this article evaluates performance using machine learning models excluding the SVC model, in which performance evaluation is inadequate. 

#### 3.2.7. Classification Step

The classification step is the final step in configuring a defense system. It is a step that classifies actual or random keyboard data from inputted data based on the results learned in the previous machine learning step. In this step, to evaluate the performance of the learned model in classifying data, indicators commonly used in machine learning performance evaluation, such as accuracy, precision, recall, F1-score, and AUC, are used. The performance indicators to be analyzed intensively in the classification step are the classification performance of the attack system and classification performance of the keyboard data defense system. 

Since the goal of the attack system is to steal actual keyboard data by classifying random keyboard data generated by the security software, the higher the classification performance, the higher the attack success rate. However, since, the defense system generated random keyboard data by using GAN to respond to prior attack techniques, the lower the classification performance, the higher the defense success rate. 

As mentioned above, we derived a methodology for the keyboard data defense technique using the proposed GAN and configured a defenses system that corresponds to the methodology. Through this, we believe that keyboard data will be safely protected from attacks that steal keyboard data. In the next Section, based on the results derived from the classification step, the performance of the prior researches as an attack system and the proposed technique as a defense system are evaluated through experiments.

## 4. Experimental Results

This section describes the results of an experimental evaluation of the performance of the proposed keyboard data defenses technique using GAN. The dataset used in the experiment is from “Section 3.2.5. Experiment and dataset configuration step”, and the experimental results were analyzed from various perspectives to prove the effectiveness of the proposed technique. 

The first perspective is to evaluate the performance related to keyboard data protection from a defense perspective of the proposed keyboard data defense technique. In particular, defense performance is evaluated by deriving keyboard data classification performance using machine learning models through random keyboard data generated by GAN. The second perspective is to evaluate the performance of the attack and defense techniques. This perspective also evaluates the performance of defense technique compared to attack technique. Finally, the third perspective is to evaluate performance by comparing the performance increase or decrease values between the attack and defense techniques. This perspective evaluates the performance of each experimental dataset using defined features by deriving the average of the increase or decrease in performance for each dataset. Moreover, the final goal of this article is to evaluate the performance of the proposed technique to decrease the keyboard attack success rate compared to prior researches. Accordingly, the performance of the proposed technique is evaluated by analyzing the number of decreases in performance. 

### 4.1. Performance Evaluation after Applying GAN

**Figure 7:**
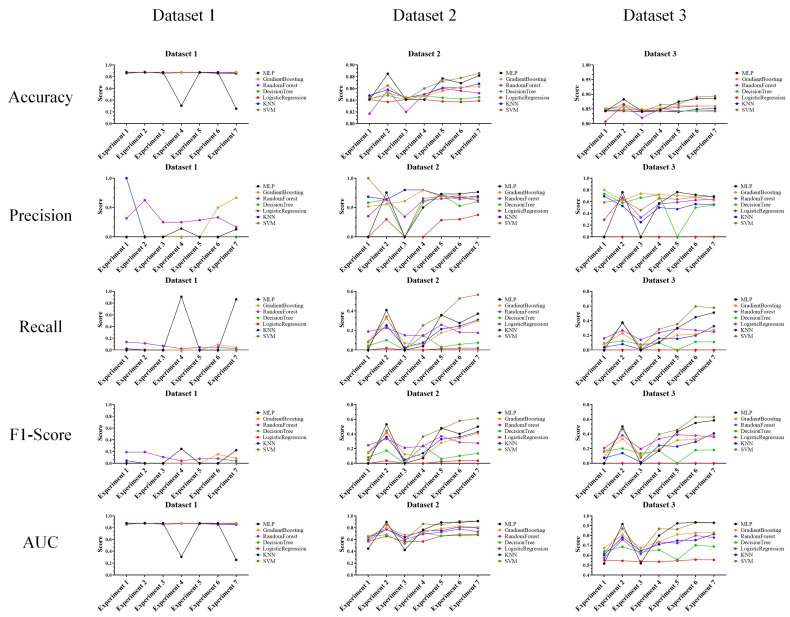
Performance evaluation results for each experiment after applying GAN.

In the performance evaluation after applying GAN, the experimental results are analyzed according to the first perspective. The overall performance evaluation results from the first perspective are shown in Figure 7.

The experimental results in the figure show the classification results of machine learning for random keyboard data and actual keyboard data generated through GAN model. Specifically, these are the performance evaluation results for accuracy, precision, recall, F1-score, and AUC each dataset for each experiment (exp. 1–7) consisting of six features. These results are found to be highly useful in cases where analysis of the performance of the keyboard data defense technique itself is required. This usefulness is because the experimental results provide an overall understanding of the data classification performance using machine learning models.

The performances of the prior research and the proposed technique are shown in the figures to compare datasets 1–3, and most of the performance of the proposed technique decreased. This result means that as the performance of machine learning classification decreases, it becomes more difficult to classify random keyboard data in keyboard data attack technique. Ultimately, even if prior research on keyboard data attack technique is used, it cannot effectively classify actual keyboard data due to the proposed technique. Next, to compare the performance in more detail with numerical values, we compare and analyze the best performances of the prior research and the proposed technique. Moreover, to understand the effectiveness of the proposed keyboard data defense technique from the defender’s perspective, the increase and decrease values are shown based on the highest performance. The results of this analysis are shown in Table 11, Table 12 and Table 13 for each dataset.

### 4.2. Performance Evaluation with Prior Researches

**Figure 8:**
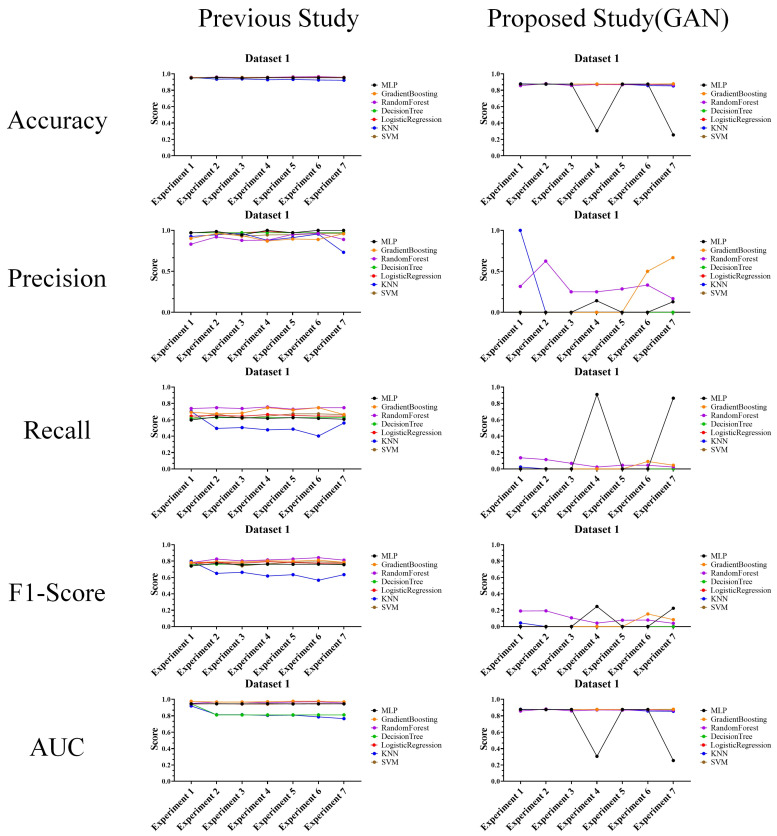
Performance evaluation results compared to prior research (Dataset 1) (**Left**: prior research, and **right**: proposed technique).

**Figure 9:**
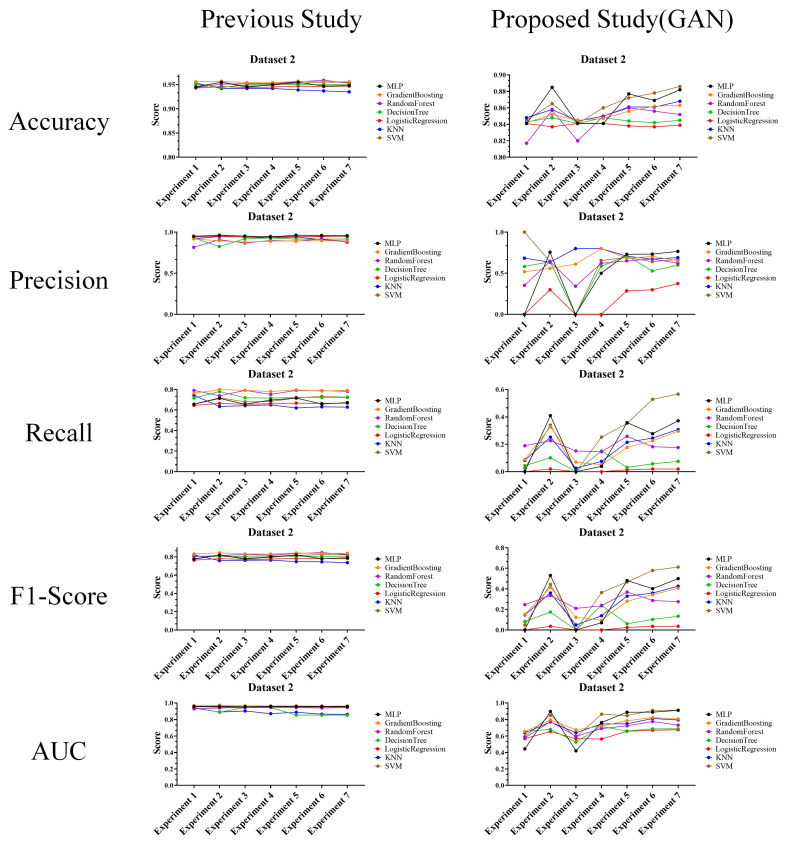
Performance evaluation results compared to prior research (Dataset 2) (**Left**: prior research, and **right**: proposed technique).

**Figure 10:**
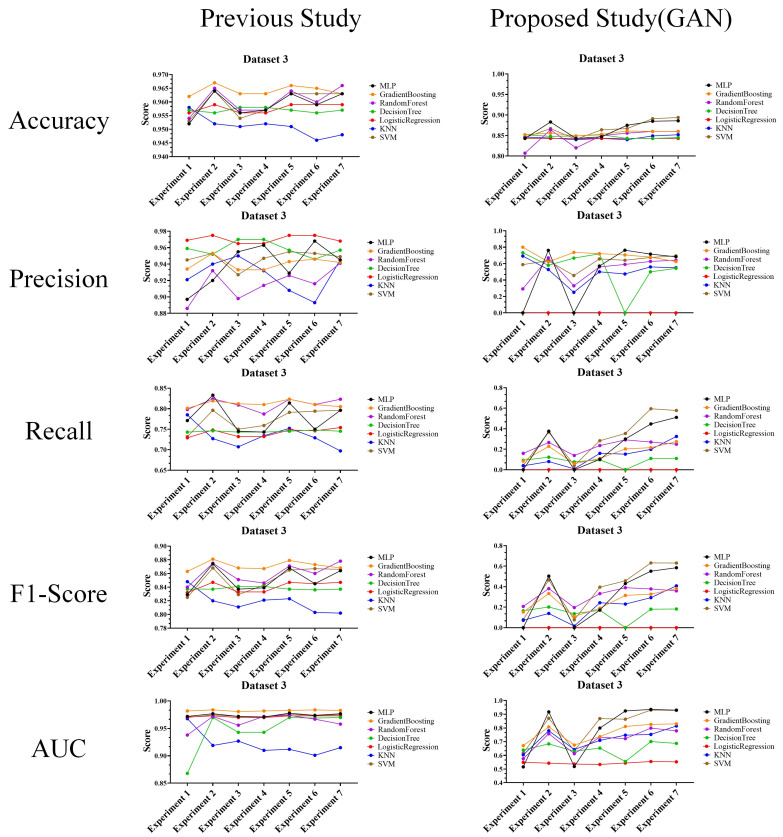
Performance evaluation results compared to prior research (Dataset 3) (**Left**: prior research, and **right**: proposed technique).

In the performance evaluation with prior researches, experimental results are analyzed according to the second perspective. The second perspective practically compares the attack and defense performances. Based on this perspective, the experimental results for each data were analyzed, and the results are shown in Figure 8, Figure 9 and Figure 10. 

The above experimental results in tables were interpreted and analyzed from the second perspective of this article and are expected to be highly useful in cases where performance comparison or analysis with prior research is required. The reason for this usefulness is that the experimental results provide features that allow comparison and evaluation of the actual performance of the attack and defense techniques. 

### 4.3. Evaluation of Performance Increase or Decrease

**Figure 11:**
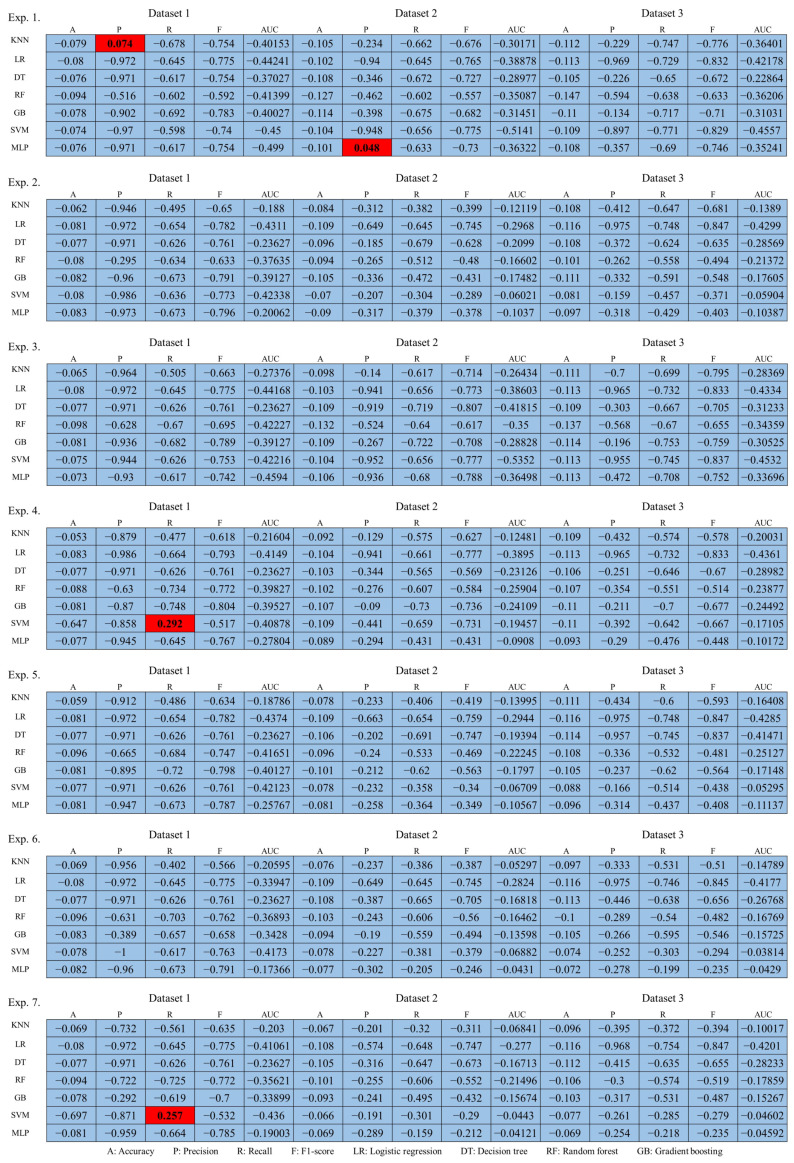
Performance increase or decrease results compared to prior researches.

In the evaluation of performance increase or decrease, the experimental results are analyzed according to the third perspective. The third perspective is to evaluate performance by comparing the performance increase or decrease values between the attack technique, which is a prior research, and the defense technique, which is a proposed technique. We analyze performance that varies depending on the ratio of random keyboard data and actual keyboard data included in the differently configured datasets for each experiment and the total amount of data. Based on this perspective, the increase or decrease in performance according to each dataset and experiment was analyzed, and the results are shown in Figure 11.

Analyzing the figure, the performance of the prior research and the proposed technique were individually compared, showing the results of performance increase or decrease according to the experiment for each dataset. The technique proposed in this article means effective defense if it decreases machine learning classification performance compared to prior researches. Therefore, compared to prior researches, area where performance decreased is expressed in blue, and conversely, areas where performance increased are expressed in red. Notably, the proposed technique was found to be most effective in Dataset 3, while Dataset 1 was found to be the least effective. In order to more intuitively show the above analysis results, the number of performance increase or decrease indicators according to the datasets and experiments is shown in Table 14.

The table shows the number of indicators with increased performance in the proposed technique compared with previous researches. As a result, the performance included in most indicators was found to be low, which means that the proposed technique effectively defends against keyboard data attack technique using machine learning. To compare performance in more detail, the overall performance of each dataset was compared based on the average of increased and decreased performances for all performance indicators, and the results are shown in Table 15.

To compare and evaluate the overall performance increase or decrease for each dataset, the experiment with the lowest decrease in performance was analyzed. From the perspective of this article, a significant decrease in machine learning performance means a significant decrease in the attack success rate, effectively protecting keyboard data. Conversely, an increase or small decrease in performance means that the keyboard data is not effectively protected, because the attack success rate increases, or the attack success rate is relatively slightly decreased. Accordingly, to select the most efficient dataset based on the experimental results, experiments with the lowest performance were expressed in red. In addition, experiments with a lot of red indicate inefficient datasets from a defense perspective. 

Analyzing the results, in Experiment 1, Dataset 2 showed the smallest decrease in performance, in that order, Dataset 2 in Experiment 2, Dataset 3 in Experiment 3, Dataset 1 in Experiment 4, and Dataset 2 in Experiment 5, Dataset 2 and Experiment 6, and finally, Dataset 1 in Experiment 7 showed the lowest performance decrease. Analyzing these results in terms of efficient dataset selection, Dataset 1 showed the lowest performance decrease in Experiments 4 and 7, Dataset 2 showed the lowest performance decrease in Experiments 1, 2, 5, and 6, and finally, Dataset 3 showed the lowest performance decrease in Experiment 3. In summary, Dataset 3 with 1 decrease is the most efficient dataset, Dataset 1 with 2 decreases is the next most efficient dataset, and finally, Dataset 2, which shows the 4 most decreases, is evaluated as the most inefficient dataset. 

**Figure 12:**
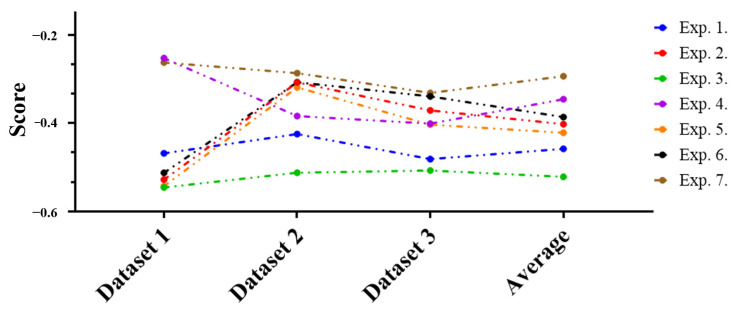
Comprehensive results of performance increase or decrease comparison with prior researches.

By combining these performance evaluation results, the overall results of performance increase or decrease are summarized and shown in Figure 12. This summary is to make it easier to understand prior research and the overall performance of the technique proposed in this article, so that all experimental results can be shown more intuitively.

The results of the summary of performance increase or decrease values for each dataset and experiment are intuitively presented through the figure. Here, when the performance increase or decrease figures are finally compared with prior researches, changes in the attack success rate can be identified. So, these changes serve as the basis for judging how effectively the keyboard data defense technique using GAN responds to the attack technique. 

First, analyzing each experiment in detail, when analyzing based on the average value of the dataset, Experiment 3 had the lowest performance, so it was evaluated as the most efficient experiment, and Experiment 1, Experiment 5, Experiment 2, Experiment 6, Experiment 4, and finally, the order of Experiment 7 was evaluated as ineffective. Analyzing the data from Figure 12 from a feature perspective shows that when defining scan code, elapsed time, scan code distance, and elapsed time-scan code Manhattan distance as features, it appears to be the most efficient response to prior keyboard data attack technique using machine learning. On the other hand, the most inefficient Experiment 7 had scan code, elapsed time, scan code distance, elapsed time-scan code Manhattan distance, elapsed time-scan code Euclidean distance (i = 1), and elapsed time-scan code Euclidean distance (i = 2) as features. 

Summarizing the experimental results overall, the keyboard data defense technique using the GAN proposed in this article decreased performance in most experiments. When defining scan code, elapsed time, scan code distance, and elapsed time-scan code Manhattan distance as features, the attack success rate decreased significantly. Based on these experimental results, we believe that if the proposed keyboard data defense technique is introduced, it will respond very effectively to the keyboard data attack technique.

## 5. Conclusions

This article proposed a keyboard data defense technique to respond to the latest keyboard data attack technique that steals user’s password by classifying random scan codes generated from security software using a machine learning model. Through experiments, we verified that keyboard data was effectively protected. The core idea of the proposed keyboard data defense technique is to cause confusion to attackers in keyboard data classification. To this end, we demonstrated that the proposed technique can respond to prior keyboard data attack techniques by using GAN model to generate random keyboard data with characteristics almost similar to actual keyboard data. 

To prove the effectiveness of the proposed technique, the experiments and datasets of prior researches were configured identically, the experimental results were analyzed from various perspectives. Moreover, the experimental results from each perspective were synthesized and the results were analyzed. To summarize the results of performance evaluation with previous research, the machine learning-based keyboard data attack, which was a prior research, showed a 96.7% attack success rate. This high success rate means almost completely classifying random keyboard data generated by the defender and actual keyboard data inputted by the user. To respond to these attacks, the technique proposed in this article showed a result of decreasing the attack success rate by about 13%. Notably, in all experiments, the average decrease in keyboard data classification performance ranged from a minimum of −29% to a maximum of 52%. When evaluating performance based on maximum performance, all performance indicators were found to decrease by more than 50%. In other words, applying the proposed keyboard data defense technique using GAN will result in very effectively lowering the attack success rate of prior data attack techniques. Due to the nature of the password-based authentication technique, if even one character is incorrect, authentication fails. Therefore, the conclusion was drawn that the response technique proposed in this article safely protects keyboard data. 

Through the results of this article, security threats due to keyboard data attack in password-based authentication can be more effectively prevented. We also believe that the results of this article can be used as a guideline for security evaluation of a password-based authentication technique.

## Figures and Tables

**Table 1:** Configuration of keyboard status register.

Bit	Description
Bit 0	OBF (Output Buffer Full)
Bit 1	IBF (Input Buffer Full)
Bit 2	System flag
Bit 3	C/D (Control/Data)
Bit 4	Inhibit switch
Bit 5	Transmit time-out
Bit 6	Receive time-out
Bit 7	Parity error

**Table 2:** Example of keyboard controller control commands (X: Parameters required; O: No parameters required).

Code	Feature	Parameter	Response
0x20	Read Configuration Register	X	Configuration register value
0x60	Write Configuration Register	O	X
0xAA	Self-Test	X	0x55
⋮	⋮		
0xC0	Read Input Port	X	Input port value
0xD0	Read Output Port	X	Output port value
0xD1	Write Output Port	O	X
**0xD2**	**Write Keyboard Output Buffer**	**O**	**Sent parameter**

**Table 3:** Example of a datasets constructed in the first prior research.

Experiment	Dataset	Feature
Exp. 1.	Dataset 1 (3522, 392/3129)	Index, Scan code
**Exp. 2.**	**Dataset 2 (10,022, 1422/8599)**	**Elapsed time, Scan code**
Exp. 3.	Dataset 3 (15,046, 281/12,764)	Elapsed time, Scan code, Flag

**Table 4:** Example of a dataset constructed in the second prior research.

Experiment	Dataset	Feature
Exp. 1.	Dataset 1 (3522, 392/3129) Dataset 2 (10,022, 1422/8599) Dataset 3 (15,046, 281/12,764)	Scan code, Elapsed time Scan code distance
Exp. 2.	Dataset 1 (3522, 392/3129) Dataset 2 (10,022, 1422/8599) Dataset 3 (15,046, 281/12,764)	Scan code, Elapsed time Scan code distance Manhattan distance between time-scan codes
Exp. 3.	Dataset 1 (3522, 392/3129) Dataset 2 (10,022, 1422/8599) Dataset 3 (15,046, 281/12,764)	Scan code, Elapsed time Scan code distance Euclidean distance between time-scan codes (i = 1)
Exp. 4.	Dataset 1 (3522, 392/3129) Dataset 2 (10,022, 1422/8599) Dataset 3 (15,046, 281/12,764)	Scan code, Elapsed time Scan code distance Euclidean distance between time-scan codes (i = 2)
Exp. 5.	Dataset 1 (3522, 392/3129) Dataset 2 (10,022, 1422/8599) Dataset 3 (15,046, 281/12,764)	Scan code, Elapsed time Scan code distance Manhattan distance between time-scan codes Euclidean distance between time-scan codes (i = 1)
Exp. 6.	Dataset 1 (3522, 392/3129) Dataset 2 (10,022, 1422/8599) Dataset 3 (15,046, 281/12,764)	Scan code, Elapsed time Scan code distance Manhattan distance between time-scan codes Euclidean distance between time-scan codes (i = 2)
Exp. 7.	Dataset 1 (3522, 392/3129) Dataset 2 (10,022, 1422/8599) Dataset 3 (15,046, 281/12,764)	Scan code, Elapsed time Scan code distance Manhattan distance between time-scan codes Euclidean distance between time-scan codes (i = 1 and i = 2)
Exp. 7.	Dataset 1 (3522, 392/3129) Dataset 2 (10,022, 1422/8599) Dataset 3 (15,046, 281/12,764)	Scan code, Elapsed time Scan code distance Manhattan distance between time-scan codes Euclidean distance between time-scan codes (i = 1 and i = 2)

**Table 5:** Summary of best performance results from second prior research (Dataset 1).

Exp.	Accuracy		Precision		Recall		F1-Score		AUC	
	Model	Best Score	Model	Best Score	Model	Best Score	Model	Best Score	Model	Best Score
1 [18]	K	0.957	L	0.972	R	0.738	K	0.798	G	0.975
2 [19]	R	0.961	M	0.986	R	0.748	R	0.825	R	0.967
3 [19]	R, G	0.956	L	0.972	R	0.738	R	0.802	R, G	0.966
4 [19]	L, R	0.958	**M**	**1**	**R**	**0.757**	R	0.814	G	0.97
5 [19]	R	0.963	L	0.972	R	0.729	R	0.825	**G**	**0.976**
6 [19]	**R**	**0.966**	**M**	**1**	R, G	0.748	**R**	**0.842**	**G**	**0.976**
7 [19]	R	0.958	**M**	**1**	R	0.748	R	0.812	G	0.968

K: KNN, L: Logistic regression, R: Random forest, G: Gradient boosting, M: MLP, S: SVM, D: Decision tree.

**Table 6:** Summary of best performance results from second prior research (Dataset 2).

Exp.	Accuracy		Precision		Recall		F1-Score		AUC	
	Model	Best Score	Model	Best Score	Model	Best Score	Model	Best Score	Model	Best Score
1 [18]	G	0.956	S	0.952	R	0.791	G	0.833	G	0.968
2 [19]	G	0.957	**M**	**0.963**	**G**	**0.799**	G	0.844	**G**	**0.972**
3 [19]	G	0.954	M	0.952	R, G	0.791	G	0.832	G	0.964
4 [19]	G	0.954	S	0.95	G	0.78	G	0.831	G	0.963
5 [19]	G	0.957	**M**	**0.963**	G	0.796	G	0.843	G	0.965
6 [19]	**R**	**0.959**	M	0.96	R	0.788	**R**	**0.847**	G	0.961
7 [19]	G	0.956	M	0.957	G	0.791	G	0.839	G	0.964

K: KNN, L: Logistic regression, R: Random forest, G: Gradient boosting, M: MLP, S: SVM, D: Decision tree.

**Table 7:** Summary of best performance results from second prior research (Dataset 3).

Exp.	Accuracy		Precision		Recall		F1-Score		AUC	
	Model	Best Score	Model	Best Score	Model	Best Score	Model	Best Score	Model	Best Score
1 [18]	G	0.962	L	0.969	G	0.801	G	0.863	G	0.982
2 [19]	**G**	**0.967**	**L**	**0.975**	**M**	**0.833**	**G**	**0.881**	**G**	**0.984**
3 [19]	G	0.963	D	0.97	G	0.812	G	0.868	G	0.981
4 [19]	G	0.963	D	0.97	G	0.81	G	0.867	G	0.982
5 [19]	G	0.966	**L**	**0.975**	R, G	0.823	G	0.878	G	0.983
6 [19]	G	0.965	**L**	**0.975**	R, G	0.81	G	0.873	**G**	**0.984**
7 [19]	R	0.966	L	0.968	R, G	0.823	R	0.878	G	0.983

K: KNN, L: Logistic regression, R: Random forest, G: Gradient boosting, M: MLP, S: SVM, D: Decision tree.

**Table 8:** Example of input and output data for GAN model.

Index	Input Data (Actual Keyboard Data)		Output Data (Random Keyboard Data)	
	Elapsed Time	Scan Code	Elapsed Time	Scan Code
0	0.062523	0.5	0.048599	0.44
1	0.062317	0.03	0.040192	0.51
2	0.062425	0.26	0.043335	0.38
3	0.062063	0.39	0.027847	0.3
4	0.062399	0.03	0.06369	0.39
5	0.062371	0.37	0.030075	0.48
⋮	⋮		⋮	
2278	0.092087	0.23	0.058807	0.06
2279	0.04863	0.12	0.046799	0.36
2280	0.062119	0.34	0.023514	0.5
2281	0.067932	0.08	0.018188	0.31

**Table 9:** Example of preprocessed results of generated random keyboard data.

Index	Random Data			Pre-Processed Data		
	Elapsed Time	Scan Code	Scan Code Distance	Manhattan Distance	Euclidean Distance (i = 1)	Euclidean Distance (i = 2)
0	0.048599	0.44	0	0	0.442675799	0
1	0.040192	0.51	0.07	0.078407	0.511581271	0.611485323
2	0.043335	0.38	0.13	0.133143	0.382462968	0.577981729
3	0.027847	0.3	0.08	0.095488	0.301289654	0.432909434
4	0.06369	0.39	0.09	0.125843	0.395166314	0.424906427
5	0.030075	0.48	0.09	0.123615	0.48094127	0.555797375
⋮	⋮			⋮		
2278	0.058807	0.06	0.15	0.159742	0.084013471	0.141456031
2279	0.046799	0.36	0.3	0.312008	0.363029126	0.313203272
2280	0.023514	0.5	0.14	0.163285	0.500552603	0.570205029
2281	0.018188	0.31	0.19	0.195326	0.310533095	0.558742473

**Table 10:** Datasets Configured for the Experiment.

Exp.	Dataset	Feature
Exp. 1.	Dataset 1 (392/3129) Dataset 2 (1422/8599) Dataset 3 (2281/12,764)	Scan code, Elapsed time Scan code distance
Exp. 2.	Dataset 1 (392/3129) Dataset 2 (1422/8599) Dataset 3 (2281/12,764)	Scan code, Elapsed time Scan code distance Manhattan distance between time-scan codes
Exp. 3.	Dataset 1 (392/3129) Dataset 2 (1422/8599) Dataset 3 (2281/12,764)	Scan code, Elapsed time Scan code distance Euclidean distance between time-scan codes (i = 1)
Exp. 4.	Dataset 1 (392/3129) Dataset 2 (1422/8599) Dataset 3 (2281/12,764)	Scan code, Elapsed time Scan code distance Euclidean distance between time-scan codes (i = 2)
Exp. 5.	Dataset 1 (392/3129) Dataset 2 (1422/8599) Dataset 3 (2281/12,764)	Scan code, x Elapsed time Scan code distance Manhattan distance between time-scan codes Euclidean distance between time-scan codes (i = 1)
Exp. 6.	Dataset 1 (392/3129) Dataset 2 (1422/8599) Dataset 3 (2281/12,764)	Scan code, Elapsed time Scan code distance Manhattan distance between time-scan codes Euclidean distance between time-scan codes (i = 2)
Exp. 7.	Dataset 1 (392/3129) Dataset 2 (1422/8599) Dataset 3 (2281/12,764)	Scan code, Elapsed time Scan code distance Manhattan distance between time-scan codes Euclidean distance between time-scan codes (i = 1 and i = 2)

**Table 11:** Results of comparison of increase and decrease values based on the highest performance (Dataset 1).

Exp.	ACC			PRE			REC			F1			AUC		
	MD	B	+/−	MD	B	+/−	MD	B	+/−	MD	B	+/−	MD	B	+/−
1	R	0.856	−0.094	L	0	−0.972	G	0	−0.692	G	0	−0.783	S	0.443	−0.499
2	S	0.875	−0.083	M	0.875	−0.986	G, S	0	−0.673	S	0	−0.796	L	0.515	−0.431
3	R	0.858	−0.098	L	0	−0.972	G	0	−0.682	G	0	−0.789	S	0.483	−0.459
4	M	0.306	−0.647	L	0	−0.986	G	0	−0.748	G	0	−0.804	L	0.530	−0.415
5	R	0.867	−0.096	L	0	−0.972	G	0	−0.72	G	0	−0.798	L	0.508	−0.437
6	R	0.87	−0.096	M	0	−1	R	0.045	−0.703	S	0	−0.791	M	0.527	−0.417
7	M	0.255	−0.697	L	0	−0.972	R	0.023	−0.725	S	0	−0.785	M	0.508	−0.436

ACC: Accuracy, PRE: Precision, REC: Recall, F1: F1-score, MD: Model, B: Best score, K: KNN, L: Logistic regression, R: Random forest, G: Gradient boosting, M: MLP, S: SVM, D: Decision tree.

**Table 12:** Results of comparison of increase and decrease values based on the highest performance (Dataset 2).

Exp.	ACC			PRE			REC			F1			AUC		
	MD	B	+/−	MD	B	+/−	MD	B	+/−	MD	B	+/−	MD	B	+/−
1	R	0.807	−0.147	L	0	−0.969	M	0	−0.771	L	0	−0.832	M	0.516	−0.456
2	L	0.843	−0.116	L	0	−0.975	L	0	−0.748	L	0	−0.847	L	0.543	−0.430
3	R	0.82	−0.137	L	0	−0.965	G	0.059	−0.753	M	0	−0.837	M	0.519	−0.453
4	L	0.843	−0.113	L	0	−0.965	L	0	−0.732	L	0	−0.833	L	0.534	−0.436
5	L	0.843	−0.116	L	0	−0.975	L	0	−0.748	L	0	−0.847	L	0.545	−0.428
6	L	0.843	−0.116	L	0	−0.975	L	0	−0.746	L	0	−0.845	L	0.555	−0.418
7	L	L	−0.116	L	0	−0.968	L	0	−0.754	L	0	−0.847	L	0.553	−0.420

ACC: Accuracy, PRE: Precision, REC: Recall, F1: F1-score, MD: Model, B: Best score, K: KNN, L: Logistic regression, R: Random forest, G: Gradient boosting, M: MLP, S: SVM, D: Decision tree.

**Table 13:** Results of comparison of increase and decrease values based on the highest performance (Dataset 3).

Exp.	ACC			PRE			REC			F1			AUC		
	MD	B	+/−	MD	B	+/−	MD	B	+/−	MD	B	+/−	MD	B	+/−
1	R	0.856	−0.094	L	0	−0.972	G	0	−0.692	G	0	−0.783	S	0.443	−0.499
2	S	0.875	−0.083	M	0.875	−0.986	G, S	0	−0.673	S	0	−0.796	L	0.515	−0.431
3	R	0.858	−0.098	L	0	−0.972	G	0	−0.682	G	0	−0.789	S	0.483	−0.459
4	M	0.306	−0.647	L	0	−0.986	G	0	−0.748	G	0	−0.804	L	0.530	−0.415
5	R	0.867	−0.096	L	0	−0.972	G	0	−0.72	G	0	−0.798	L	0.508	−0.437
6	R	0.87	−0.096	M	0	−1	R	0.045	−0.703	S	0	−0.791	M	0.527	−0.417
7	M	0.255	−0.697	L	0	−0.972	R	0.023	−0.725	S	0	−0.785	M	0.508	−0.436

ACC: Accuracy, PRE: Precision, REC: Recall, F1: F1-score, MD: Model, B: Best score, K: KNN, L: Logistic regression, R: Random forest, G: Gradient boosting, M: MLP, S: SVM, D: Decision tree.

**Table 14:** Comparison results of the number of performance increase or decrease indicators according to datasets and experiments.

Experiment	+/−	Dataset 1	Dataset 2	Dataset 3
Exp. 1.	Decrease	34	34	35
	Increase	**1**	**1**	0
Exp. 2.	Decrease	35	35	35
	Increase	0	0	0
Exp. 3.	Decrease	35	35	35
	Increase	0	0	0
Exp. 4.	Decrease	34	35	35
	Increase	**1**	0	0
Exp. 5.	Decrease	35	35	35
	Increase	0	0	0
Exp. 6.	Decrease	35	35	35
	Increase	0	0	0
Exp. 7.	Decrease	34	35	35
	Increase	**1**	0	0

**Table 15:** Average comparison results of performance increase or decrease items for each dataset.

Experiment	Dataset 1	Dataset 2	Dataset 3	Average
Exp. 1.	−0.46828	**−0.4246**	−0.48128	−0.45806
Exp. 2.	−0.52777	**−0.3078**	−0.3712	−0.40227
Exp. 3.	−0.54542	−0.51203	**−0.5071**	−0.52151
Exp. 4.	**−0.25270**	−0.38386	−0.40096	−0.34583
Exp. 5.	−0.54235	**−0.3190**	−0.40329	−0.42154
Exp. 6.	−0.51221	**−0.3074**	−0.33929	−0.3863
Exp. 7.	**−0.26260**	−0.28682	−0.33142	−0.29361

## Data Availability

The data presented in this study are available on request from the corresponding author. The data are not publicly available due to privacy.
